# ID 232 - Avaliação da Qualidade Metodológica de Diretrizes Clínicas para Prática Odontológica na Atenção Primária à Saúde

**DOI:** 10.5327/2237-9622.2025.v34s1.232

**Published:** 2025-11-25

**Authors:** Julia Galato Rosso, Dayani Galato

**Keywords:** guia de prática clínica, traumatismos dentários, estudo de avaliação, Atenção Primária à Saúde, prática clínica baseada em evidência

## Abstract

**Introdução:**

Com o intuito de melhorar as práticas de cirurgiões-dentistas na Atenção Primária à Saúde (APS), estão sendo desenvolvidas, pelo e pela Coordenação-Geral de Saúde Bucal vinculada ao Ministério da Saúde, diretrizes baseadas em evidências, cujos primeiros documentos foram publicados no primeiro semestre de 2024. Esses documentos são apresentados nas versões estendida e resumida. Este trabalho tem como objetivo realizar a avaliação da qualidade metodológica das diretrizes relacionadas aos traumatismos alveolodentários em dentes permanentes e decíduos.

**Método:**

Trata-se de um estudo descritivo com aplicação da ferramenta (AGREE II) por dois avaliadores das diretrizes de manejo clínico de traumatismos alveolodentários em dentes permanentes (versão estendida e resumida) e em dentes decíduos (versão resumida e estendida). Para cada um dos 23 itens do AGREE II, cada avaliador atribuiu um entre 1 (discordo totalmente) e 7 (concordo completamente). Esses itens estavam divididos em seis domínios: escopo e propósito; envolvimento das partes interessadas; rigor no desenvolvimento; clareza da apresentação; aplicabilidade; e independência editorial. As pontuações globais foram estimadas pela média (MD) e pelo desvio-padrão (DP). Para avaliar a diferença entre os, usou-se o teste t de Student, considerando p<0,05 como significante.

**Resultados:**

A diretriz na versão resumida relacionada aos traumatismos alveolodentários em dentes permanentes obteve menor pontuação global (MD=3,95, DP=2,85) do que a versão estendida (MD=6,02, DP=2,23), demonstrando uma diferença significativa (p=0,009). O mesmo foi observado em relação à diretriz resumida para dentes decíduos (MD=3,87, DP=2,80) e à estendida (MD=6,00, DP=2,23), mostrando-se também diferente (p=0,007). As diferenças ocorreram principalmente na avaliação do domínio rigor no desenvolvimento. Mesmo que as versões estendidas tenham recebido maior pontuação e que tenham listados os autores envolvidos, não há informações explícitas sobre o envolvimento das partes interessadas, pacientes/responsáveis e profissionais da Atenção Primária. Ainda, destaca-se que não foi identificado um procedimento de atualização das diretrizes avaliadas, bem como não há a descrição de facilitadores e dificultadores da sua aplicação nem são apresentados os critérios para seu monitoramento e sua auditoria.

**Conclusão:**

Este trabalho representa a primeira avaliação metodológica das diretrizes de manejo clínico de traumatismos alveolodentários na Atenção Primária analisadas por meio do AGREE II. Os achados demonstram o rigor metodológico adotado. A versão resumida, mesmo tendo menor pontuação global, representa uma ferramenta importante para disseminação de boas práticas relacionadas ao manejo de traumatismo alvéolodentário na Atenção Primária. Sugere-se maior transparência, principalmente em relação ao processo de atualização e de aplicação das diretrizes.

